# Exhaustive drainage versus fixed-time drainage for chronic subdural hematoma after one-burr hole craniostomy (ECHO): study protocol for a multicenter randomized controlled trial

**DOI:** 10.1186/s13063-023-07250-y

**Published:** 2023-03-20

**Authors:** Liang Wu, Yunwei Ou, Bingcheng Zhu, Xufei Guo, Xiaofan Yu, Long Xu, Jinping Li, Enshan Feng, Huaqing Li, Xiaodong Wang, Huaqun Chen, Zhaosheng Sun, Zaofu Liu, Dawei Yang, Hongbing Zhang, Zhigang Liu, Jie Tang, Shangfeng Zhao, Guobin Zhang, Jiemin Yao, Dongming Ma, Zelin Sun, Hui Zhou, Baiyun Liu, Weiming Liu, ECHO Trial Collaborators

**Affiliations:** 1 Department of Neurosurgery, Beijing Tiantan Hospital, Capital Medical University, Beijing, China; 2 China National Clinical Research Center for Neurological Diseases, Beijing, China; 3 Department of Neurosurgery, Beijing Chaoyang Hospital, Capital Medical University, Beijing, China; 4 Department of Neurosurgery, Beijing Ditan Hospital, Capital Medical University, Beijing, China; 5 Department of Neurosurgery, Xinxing County People’s Hospital, Yunfu, Guangdong China; 6 Department of Neurosurgery, Puning People’s Hospital, Puning, Guangdong China; 7 Department of Neurosurgery, Yancheng Third People’s Hospital, Yancheng, Jiangsu China; 8 Department of Neurosurgery, Hengshui People’s Hospital, Hengshui, Hebei China; 9 Department of Neurosurgery, Wei County Hospital of Traditional Chinese Medicine, Handan, Hebei China; 10 Department of Neurosurgery, First Hospital of Qinhuangdao, Qinhuangdao, Hebei China; 11 Department of Neurosurgery, Beijing Luhe Hospital, Capital Medical University, Beijing, China; 12 Department of Neurosurgery, Xiahuayuan District Hospital, Zhangjiakou, Hebei China; 13 Department of Neurosurgery, Beijing Xuanwu Hospital, Capital Medical University, Beijing, China; 14 Department of Neurosurgery, Beijing Tongren Hospital, Capital Medical University, Beijing, China; 15 Department of Neurosurgery, Tianjin Huanhu Hospital, Tianjin, China; 16 Department of Neurosurgery, The Second Nanning People’s Hospital, Nanning, Guangxi China; 17 Department of Neurosurgery, People’s Hospital of Ningxia Hui Autonomous Region, Yinchuan, Ningxia China; 18 Department of Neurosurgery, North China University of Science and Technology Affiliated Hospital, Tangshan, Hebei China; 19 Department of Neurosurgery, First People’s Hospital of Lianyungang, Lianyungang, Jiangsu China

**Keywords:** Chronic subdural hematoma, Exhaustive drainage, Fixed-time drainage, Burr-hole craniostomy, Recurrence, Protocol, Randomized controlled trial

## Abstract

**Background:**

Chronic subdural hematomas (CSDHs) are one of the most common neurosurgical conditions. The standard surgical technique includes burr-hole craniostomy, followed by intraoperative irrigation and placement of subdural closed-system drainage. The drainage is generally removed after 48 h, which can be described as fixed-time drainage strategy. According to literature, the recurrence rate is 5–33% with this strategy. In our retrospective study, postoperative hematoma volume was found to significantly increase the risk of recurrence. Based on these results, an exhaustive drainage strategy is conducted to minimize postoperative hematoma volume and achieve a low recurrence rate and good outcomes.

**Methods:**

This is a prospective, multicenter, open-label, blinded endpoint randomized controlled trial designed to include 304 participants over the age of 18–90 years presenting with a symptomatic CSDH verified on cranial computed tomography or magnetic resonance imaging. Participants will be randomly allocated to perform exhaustive drainage (treatment group) or fixed-time drainage (control group) after a one-burr hole craniostomy. The primary endpoint will be recurrence indicating a reoperation within 6 months.

**Discussion:**

This study will validate the effect and safety of exhaustive drainage after one-burr hole craniostomy in reducing recurrence rates and provide critical information to improve CSDH surgical management.

**Trial registration:**

Clinicaltrials.gov, NCT04573387. Registered on October 5, 2020.

## Administrative information

**Table:** 

Title {1}	Exhaustive drainage versus fixed-time drainage for chronic subdural hematoma after one-burr hole craniostomy (ECHO): study protocol for a multicenter randomized controlled trial
Trial registration {2a and 2b}	Clinicaltrials.gov, NCT04573387. Registered on October 5, 2020. https://www.clinicaltrials.gov/ct2/show/study/NCT04573387
Protocol version {3}	2020/07/31 Protocol Version 1.0
Funding {4}	Capital Medical Development and Research Fund (grant No.2020–2-2045)
Author details {5a}	Liang Wu, Department of Neurosurgery, Beijing Tiantan Hospital, Capital Medical University, Beijing, China Yunwei Ou, Department of Neurosurgery, Beijing Tiantan Hospital, Capital Medical University, Beijing, China Bingcheng Zhu, Department of Neurosurgery, Beijing Tiantan Hospital, Capital Medical University, Beijing, China Xufei Guo, Department of Neurosurgery, Beijing Tiantan Hospital, Capital Medical University, Beijing, China Xiaofan Yu, Department of Neurosurgery, Beijing Tiantan Hospital, Capital Medical University, Beijing, China Long Xu, Department of Neurosurgery, Beijing Tiantan Hospital, Capital Medical University, Beijing, China Jinping Li, Department of Neurosurgery, Beijing Chaoyang Hospital, Capital Medical University, Beijing, China Enshan Feng, Department of Neurosurgery, Beijing Ditan Hospital, Capital Medical University, Beijing, China Huaqing Li, Department of Neurosurgery, Xinxing County People's Hospital, Yunfu, Guangdong, China Xiaodong Wang, Department of Neurosurgery, Puning People's Hospital, Puning, Guangdong, China Huaqun Chen, Department of Neurosurgery, Yancheng Third People's Hospital, Yancheng, Jiangsu, China Zhaosheng Sun, Department of Neurosurgery, Hengshui People's Hospital, Hengshui, Hebei, China Zaofu Liu, Department of Neurosurgery, Wei County Hospital of Traditional Chinese Medicine, Handan, Hebei, China Dawei Yang, Department of Neurosurgery, First Hospital of Qinhuangdao, Qinhuangdao, Hebei, China Hongbing Zhang, Department of Neurosurgery, Beijing Luhe Hospital, Capital Medical University, Beijing, China Zhigang Liu, Department of Neurosurgery, Xiahuayuan District Hospital, Zhangjiakou, Hebei, China Jie Tang, Department of Neurosurgery, Beijing Xuanwu Hospital, Capital Medical University, Beijing, China Shangfeng Zhao, Department of Neurosurgery, Beijing Tongren Hospital, Capital Medical University, Beijing, China Guobin Zhang, Department of Neurosurgery, Tianjin Huanhu Hospital, Tianjin, China Jiemin Yao, Department of Neurosurgery, The Second Nanning People's Hospital, Nanning, Guangxi, China Dongming Ma, Department of Neurosurgery, People's Hospital of Ningxia Hui Autonomous Region, Yinchuan, Ningxia, China Zelin Sun, Department of Neurosurgery, North China University of Science and Technology Affiliated Hospital, Tangshan, Hebei, China Hui Zhou, Department of Neurosurgery, First People's Hospital of Lianyungang, Lianyungang, Jiangsu, China Baiyun Liu, Department of Neurosurgery, Beijing Tiantan Hospital, Capital Medical University, Beijing, China Weiming Liu, Department of Neurosurgery, Beijing Tiantan Hospital, Capital Medical University, Beijing, China
Name and contact information for the trial sponsor {5b}	Beijing Municipal Health Commission Contact information: 008,601,083,970,601
Role of sponsor {5c}	The funders have no role in study design; collection, management, analysis, and interpretation of data; writing of the report; and the decision to submit the report for publication manuscript

## Introduction

### Background and rationale {6a}

Chronic subdural hematomas (CSDHs) are one of the most common neurosurgical conditions. The goal of surgery is to alleviate symptoms and minimize the risk of symptomatic recurrences. The common surgical technique includes burr-hole craniostomy, followed by intraoperative irrigation and placement of subdural closed-system drainage. The drainage is generally removed after 48 h, which can be described as a fixed-time drainage strategy [1]. According to literature, the recurrence rate is 5–33% with this strategy [2–8]. Therefore, the optimal standard management procedure for CSDH remains uncertain. In our retrospective study, postoperative hematoma volume (*p* = 0.001, *B* = 0.028, Exp(*B*) = 1.028, 95% CI 1.011–1.046) was found to significantly increase the risk of recurrence [9]. Based on these results, an exhaustive drainage strategy is described to minimize postoperative hematoma volume and achieve a low recurrence rate and good outcomes.

In this treatment strategy, all patients were treated with a one-burr-hole craniostomy with irrigation and a closed drainage system. If the computed tomography (CT) scan on the first day after surgery (postoperative 24 ± 4 h) indicated that no obvious hematoma was left (hematoma showed no high density and had less than 3 mm of maximum width in axial CT scan images), the drainage catheter was removed when drainage ceased. If subdural collections remained in the hematoma cavity, the patient was treated with urokinase injection into the hematoma cavity through the catheter. The catheter was closed and reopened in 2 h, and a CT scan was performed after 24 h. If the CT scan showed no residual hematoma left, the catheter was removed when drainage ceased. However, if there was still an obvious residual hematoma, the above steps were repeated, and the patient was subjected to urokinase injection until the subdural collection was eliminated and drainage ceased.

In our experience, a total of 1126 patients with CSDH were treated with this exhaustive drainage strategy. The recurrence rate was 1.9% (21/1117), and 97.0% (1092/1117) of patients had good outcomes (modified Rankin Scale, mRS scores 0–3) at 6 months after discharge. Therefore, for evidence-based recommendations concerning CSDH management, a randomized controlled trial is needed to validate our previous results for the exhaustive drainage strategy.

### Objectives {7}

We hypothesize that compared with fixed-time drainage, exhaustive drainage after one-burr hole craniostomy reduces recurrence rates and improves clinical outcomes at 6 months in patients with CSDH. Consequently, the defined null hypothesis will be that there is no difference between the groups.

### Trial design {8}

**Fig. 1:**
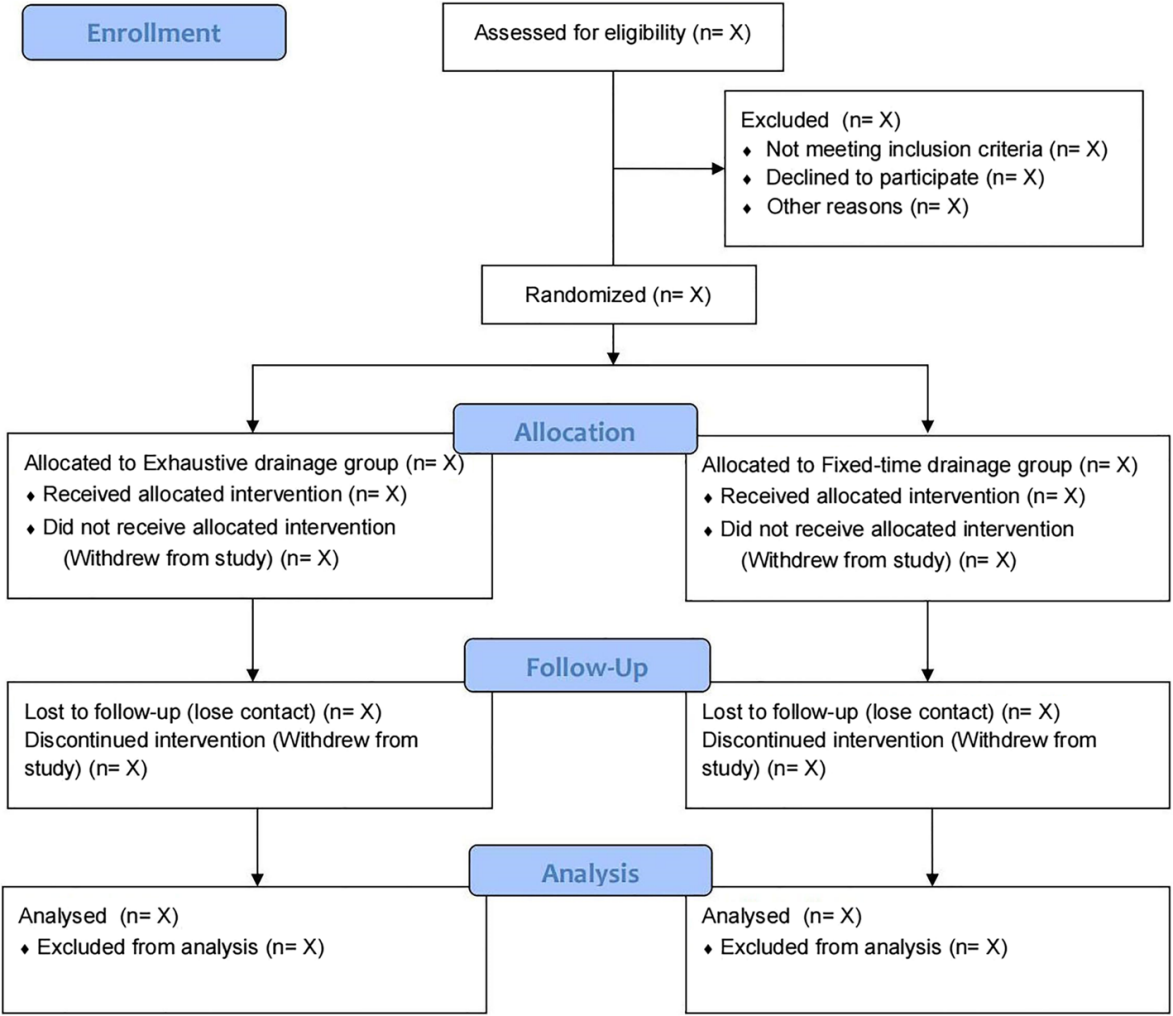
Consolidated Standards of Reporting Trials (CONSORT) patient flow diagram

The ECHO trial is a prospective, randomized, open-labeled, blinded endpoint, multicenter clinical study designed to compare differences of recurrence rate and clinical outcome from operation up to 6 months postoperatively between the exhaustive drainage group and fixed-time drainage group. In total, 304 patients will be randomly assigned to the exhaustive drainage (treatment) group and fixed-time drainage (control) group at a 1:1 ratio. The Consolidated Standards of Reporting Trials (CONSORT) patient flow diagram is presented in Fig. 1.

### Methods: participants, interventions, and outcomes

#### Study setting {9}

This is a multicenter study that will be conducted from December 2020 to May 2023 at 19 hospitals in China. In the participating centers, all patients who are diagnosed with a CSDH are tested for eligibility for the ECHO study. After gaining informed consent, patients are randomized to either the treatment or the control group. Research is facilitated by a leader center to guarantee compliance with protocol, adherence to International Conference on Harmonization-Good Clinical Practice (ICH-GCP), and data safety.

### Eligibility criteria {10}

#### Inclusion criteria

1. Patient (18 years to 90 years) presenting with clinical symptoms and neurological deficits of chronic subdural hematoma.
2. Chronic subdural hematoma verified on cranial CT or magnetic resonance imaging.
3. Written informed consent from patients or their next of kin according to the patient’s cognitive status.

#### Exclusion criteria

1. No clinical symptoms correlating with chronic subdural hematoma.
2. Lack of mass effect, and no need for surgery judged clinically by neurosurgeons.
3. Previous surgery for chronic subdural hematoma during the past 6 months.
4. Previous intracranial surgery for any neurological disorders but chronic subdural hematoma before.
5. Existing poor medication condition or severe comorbidity so that surgery cannot be tolerated, or follow-up cannot be completed.
6. Severe coagulopathy or high risk of life-threatening bleeding.
7. Postoperative cooperation is suspected to be insufficient for follow-up for 6 months.
8. Reproductive-age women without verified negative pregnancy testing.
9. Participating in another research.

### Who will take informed consent? {26a}

Informed consent will be obtained from all participants in the study prior to surgery of the CSDH. Based on the inclusion and exclusion criteria, patients will be screened for study participation. A verbal explanation of the written consent will be provided by the attending neurosurgeon, and any questions regarding the rationale, design, risks, and potential benefits of the study will be answered. Each participant will have sufficient time to decide whether to participate in this study. If the patient is unable to give consent, consent will be sought from a close relative. Withdrawal from the study is possible at any time, in accordance with the latest version of the declaration of Helsinki 2013.

### Additional consent provisions for collection and use of participant data and biological specimens {26b}

On the consent form, participants will be asked for permission for the research team to use their data and share them with people from relevant regulatory authorities. For this study, no biological specimens will be collected for storage.

### Interventions

#### Explanation for the choice of comparators {6b}

All participants will be treated with a one-burr-hole craniostomy with irrigation and a closed drainage system. In fixed-time drainage (control group), the drainage will be removed after 48 h postoperatively. In exhaustive drainage (treatment group), the drainage will be removed when postoperative hematoma volume is minimized with repeated urokinase injection into the hematoma cavity through a catheter based on a postoperative CT scan.

### Intervention description {11a}

#### Operation

All participants are treated with burr-hole craniostomy and a drainage system as follows. Participants undergo the surgical procedure under local anesthesia with a lateral position, and general anesthesia is performed if the participant cannot tolerate the surgery. A single 1.5-cm burr hole is drilled over the maximum width of the hematoma cavity. After coagulating with bipolar diathermy, the dura mater is opened with a cruciate incision. A soft catheter is placed carefully in all directions of the hematoma cavity for irrigating with 1000 mL warm Ringer’s lactate saline until clarification of subdural collections. The catheter is placed ½ length of the maximum diameter of the hematoma cavity toward the frontal region. After the skin is closed, the catheter is connected to a collection bag which is placed 15 cm under the head for passive drainage. During the drainage period, participants stay in bed until the drain is removed.

### Postoperative drainage strategy

In the fixed-time drainage group, the drainage will be removed after 48 h postoperatively. In the exhaustive drainage group, if the CT scan on the first day after surgery (postoperative 24 ± 4 h) indicates no obvious hematoma left(hematoma shows no high density and has less than 3 mm of maximum width in axial CT scan images), the drainage catheter will be removed when drainage ceases. If subdural collections remain in the hematoma cavity (hematoma shows high density and has more than 3 mm of maximum width in axial CT scan images), the participant will be treated with 30,000U urokinase injection into the hematoma cavity through the catheter. The catheter will be closed and reopened in 2 h, and a CT scan will be performed when drainage ceases. If the CT scan shows residual hematoma eliminated, the catheter will be removed. However, if there is still a residual subdural collection, the above steps will be repeated. If the participant is subjected to urokinase injection for 3 times, the catheter will be removed when drainage ceases.

### Postoperative follow-up CT

All participants will undergo a CT scan before the drainage is removed, before the patient is discharged from the hospital, and at 1, 3, and 6 months after surgery, retrospectively.

### Criteria for discontinuing or modifying allocated interventions {11b}

The allocated interventions cannot be modified during study participation, and crossover to the other study arm is not allowed. Cessation of the treatment is mandatory if a suspected serious adverse event (SAE) occurs, or participants request to withdraw from the study. Participants can withdraw their consent at any time and terminate their participation in the study prematurely. Withdrawal from the study and detailed reasons will be recorded if known. Moreover, the principal investigator (PI) is entitled to terminate the study prematurely if patient recruitment remains inadequate, or unacceptable risks arise after assessment by the Data Safety Monitoring Board (DSMB).

A detailed recording of all the adverse events (AEs) during the study will be closely monitored, and reported to the ethics committee as soon as possible, with the intention of a resolution or stabilization, or even termination of the study if necessary.

### Strategies to improve adherence to interventions {11c}

Study treatment takes place after enrollment, and follow-up visits take place at 1, 3, and 6 months. At enrollment, participants will receive detailed instructions about burr-hole craniostomy and the corresponding postoperative drainage strategy. Moreover, the importance of adherence to the study protocol is emphasized.

During follow-up visits at 1, 3, and 6 months, treatment adherence is monitored by asking whether the participant experiences any side effects or AEs. Study participants can contact one of the researchers with any questions during the study period. The whole study process is continuously monitored by a blinded local researcher in each center who performs the follow-up outcome measurements.

### Relevant concomitant care permitted or prohibited during the trial {11d}

Besides the study intervention, patients in both groups are treated according to the currently established standard of care at the trial center. All patients receive a standard operation and standard postoperative care. Interventions to postoperative drainage are prohibited. Any other medical treatments as parts of routine clinical practice are permitted during study participation.

### Provisions for post-trial care {30}

All study participants will still receive standard care and extended follow-up after the study is ended, and they can claim reimbursement from the study insurance to compensate for trial-associated harm.

### Outcomes {12}

#### Primary outcome

The primary outcome is a recurrence rate of CSDH up to 6 months after surgery. Recurrence is defined as the occurrence of symptoms and signs attributable to an ipsilateral hematoma seen on a CT scan within 6 months of the original drainage procedure.

#### Secondary outcome

Secondary outcomes include functional outcome (modified Rankin Scale, mRS score [10] and Markwalder Grading Scale, MGS grade [11]) at baseline, and at 1, 3, and 6 months; quality of life (five-dimensional EuroQol, EQ-5D-5L [12]) at baseline, and at 1, 3, and 6 months; mortality at 6 months; and rate of complications and adverse events between groups within 6 months.

### Participant timeline {13}

Participants in the intervention and control groups will undergo six scheduled follow-up visits, respectively. The participant timeline is shown in Table 1.

**Table 1:** Schedule of enrollment, interventions, and assessments

	**Enrolment**	**Surgery**	**After surgery**			
**Time point**	**Ad**	**IO**	**D1**	**1 mon**	**3 mon**	**6 mon**
**Enrollment**						
Eligibility screen	X					
Informed consent	X					
Randomization	X					
**Interventions**						
Exhaustive or fixed-time (48 h) drainage		X	X			
**Assessments**						
Demographics, medical history	X					
Laboratory data	X		X	X	X	X
Neurological examination	X		X	X	X	X
Computed tomography	X		X^#^	X	X	X
mRS	X			X	X	X
MGS	X			X	X	X
EQ-5D-5L	X			X	X	X
Complications		X	X	X	X	X
Mortality				X	X	X
Economic data						X
(S) AEs		X	X	X	X	X

*Ad* admission, D day, *EQ-5D-5L* EuroQoL 5-Dimension 5-Level questionnaire, *h* hours, *IO* intraoperative, *mon* months, *MGS* Markwalder Grading Scale, *mRS* modified Rankin scale, *(S) AE* (severe) adverse event

^#^Only in exhaustive drainage group

### Sample size {14}

Recurrent CSDH is considered both a clinically relevant and methodologically reliable, objective primary trial endpoint. In one of the largest studies to date, Santarius et al. compared a prospective series of 215 CSDH patients with randomly undergoing drains, which was removed after 48 h, or no drains after burr-hole evacuation [3]. Recurrence occurred in 10 of 108 (9.3%) people in the drainage group. According to literature, the recurrence rate is 5–33% with this strategy.

In view of the results of published studies (in particular Santarius et al.) and our retrospective series, we conservatively presume recurrence rates of 10% in the control (fixed-time drainage) and 2% in the experimental (exhaustive drainage) arm during an observation period of 6 months. For a power of 80% and a total alpha of 5%, data from 276 patients (138 per group) are needed to detect a risk difference of 20% by a *z*-test for independent samples. Assuming a drop-out and lost-to-follow-up rate of 10%, we plan to enroll 304 patients (152 per group) unless the interim look prompts any adjustment.

### Recruitment {15}

The ECHO trial will recruit in 19 hospitals in China, and there are two research members from the department of neurosurgery in each study site who will be in charge of the patient recruitment process.

### Assignment of interventions: allocation

#### Sequence generation {16a}

Eligible participants will be randomly assigned by a computerized random-number list generator used for randomization in a web-based, GCP-compliant electronic data capture (EDC) system, after written consent is obtained. Randomization is done separately in each center.

#### Concealment mechanism {16b}

Participants are randomized using the EDC system for collecting patients’ data in clinical trials. To ensure allocation concealment, the system will release the randomization code after the patient has been recruited into the trial. Participants who give consent to participate and who fulfill the inclusion criteria will be recruited by the neurosurgeons involved in the ECHO trial. The randomization will be firstly released after the signed consent form is uploaded to the system.

#### Implementation {16c}

An experienced sub-investigator, not involved in any other aspect of this study, will use the online EDC system to generate a computerized random-number list, which will allocate participants to either one of the two groups. Extended stratified block algorithms generate an unpredictable allocation sequence. Random assignment cannot be influenced by clinical investigators.

### Assignment of interventions: blinding

#### Who will be blinded {17a}

The investigators in charge of the postoperative follow-up evaluation will be blinded, along with the outcome assessors and data analysts.

#### Procedure for unblinding if needed {17b}

The design is open labeled with only the investigators for postoperative follow-up evaluation, the outcome assessors, and data analysts being blinded, so unblinding will not occur. Before the outcome assessment begins at every follow-up evaluation, the patients will be reminded not to reveal any information about their group allocation for decreasing the risk of unblinding. If details of group allocation can be detected by the investigator during follow-ups, another blinded researcher will replace to evaluate the outcome.

### Data collection and management

#### Plans for assessment and collection of outcomes {18a}

The outcome of interest will be collected by a group of blinded research members in charge of the follow-up evaluation and be recorded in the EDC system, which will only be accessible to the sub-investigators. At the completion of the 6-month follow-up data collection, we will perform a data quality audit. An investigator will sample every participant file and check for missing data.

#### Plans to promote participant retention and complete follow-up {18b}

Follow-up will be conducted on 1, 3, and 6 months after discharge by an experienced research member blinded to the study. All the participants will complete a 6-month follow-up. Follow-up data collection will be done in person during the patient follow-up visits. Any participants who do not complete the entire 6-month follow-up process due to deviation from intervention, discontinuation for personal reasons, or failure of contact, will not be replaced by other patients. Participants will be allowed to withdraw their consent or discontinue participation without any restriction, at any time throughout the study and further data associated with the trial will be collected.

### Data management {19}

All data collected will be stored in an online EDC system accessible only to investigators. Primary and secondary outcomes will be collected by a group of blinded research members in charge of the postoperative follow-up evaluation. At the completion of the 6-month follow-up data collection, a data quality audit will be performed.

### Confidentiality {27}

All personal information about the participants will be collected and stored in a secure EDC system, throughout the duration of the study, to guarantee confidentiality. All participants will be allocated by individual trial identification numbers. Only the lead investigator will have access to all the files corresponding to the personal data of the participants.

### Plans for collection, laboratory evaluation, and storage of biological specimens for genetic or molecular analysis in this trial/future use {33}

This trial does not involve collecting, laboratory evaluation, and storage of biological specimens for genetic or molecular analysis.

### Statistical methods

#### Statistical methods for primary and secondary outcomes {20a}

Statistical analyses will be performed using a statistical package (SPSS software 25.0). The Kolmogorov–Smirnov test will be used to assess the normality of variables. Data for normal distribution will be presented as mean ± standard error of mean. Variables for skewed distributions will be described as median and interquartile ranges. Categorical variables will be expressed as frequencies with percentages.

Comparisons between the groups will be carried out using an independent *t*-test to compare normally distributed data, the Mann–Whitney *U* test to skewed data, and *χ*^2^ test or Fisher’s exact test to compare categorical data such as safety analyses with the incidence of AEs. For numerical data collected at different time points throughout the course of 6 months, repeated measures analysis of variance will be performed between the two groups. The significance level will be set at *p* < 0.05.

#### Interim analyses {21b}

Although there are no anticipated problems that may be detrimental to the participants, serious life-threatening adverse events leading to prolonged hospital stay or death will be reported to the Institutional Review Board (IRB) and our study will be terminated immediately. There will be no interim analyses in this trial.

#### Methods for additional analyses (e.g., subgroup analyses) {20b}

Prior to statistical analysis, a sub-investigator will review the data record forms to check for their legitimacy and identify the missing data. The subgroup analysis will be conducted to evaluate outcomes in patients based on their baseline clinical and demographic characteristics such as gender, age, type of hematoma in CT scan, antithrombosis, and comorbidity.

#### Methods in analysis to handle protocol non-adherence and any statistical methods to handle missing data {20c}

All researchers will be trained referring to the same training protocol. Protocol modifications will not be expected. Missing clinical data, if any, will be obtained from the electronic hospital files. Postoperative follow-up evaluation at specified time points is mandatory and missing data are not to be anticipated. Analyses of all outcomes will be performed according to the intention-to-treat principle, and once enrolled, all participants will be analyzed, regardless of the findings.

#### Plans to give access to the full protocol, participant-level data, and statistical code {31c}

Data collected will be kept in the online EDC system. Only the research members and the IRB of all study sites will have access to the files. After the completion of the study, the results will be made public through publication in a scientific journal along with conferences related to neurosurgery, and the clinicaltrials.gov website. The data generated or analyzed during this study will be available from the corresponding author on reasonable request.

### Oversight and monitoring

#### Composition of the coordinating center and trial steering committee {5d}

The coordinating center (CC) will comprise of a PI, two neurosurgeons, and one methodologist. The role of CC is training of the research members from all study sites regarding every aspect of the study protocol including recruitment, preoperative evaluation, intervention, and follow-up evaluation, along with the coordination and supervision of all standardized data management and quality control.

The Trial Steering Committee (TSC) will include three independent neurosurgery experts and two independent statisticians. It will be responsible for recruitment and progress of the trial, overall supervision, and quality control during the study, and ensure standardized training for research members regarding the study protocol.

#### Composition of the data monitoring committee, its role and reporting structure {21a}

An independent DSMB consists of two statisticians and two clinical experts. All members are blinded and independent of the study and investigators. The DSMB will monitor the study data, participant safety, and SAEs throughout the trial and be responsible for all the statistical work. The DSMB will regularly report blinded statistical data to TSC, and subsequent meetings will be held at every 25%, 50%, 75%, and 100% of participant inclusions with interim analyses.

### Adverse event reporting and harms {22}

The IRB of all 19 study sites will supervise the trial progress annually. Any adverse events will be recorded, and a thorough assessment of the potential association between the study interventions and the adverse event will be carried out. Serious life-threatening AEs leading to prolonged hospital stay or death will be reported to the IRB and the trial will be terminated immediately.

### Frequency and plans for auditing trial conduct {23}

The IRB of all study sites will be making regular inspections of trial conduct. The inspections will be independent from the investigators and the sponsor. Data management researcher will stay in regular contact with the investigators about trial progress, data consistency, and follow-up visit violations.

### Plans for communicating important protocol amendments to relevant parties (e.g., trial participants, ethical committees) {25}

Although changes or amendments are not to be expected, any trial deviations from the present protocol will be fully documented using a breach report form. Protocol amendments will be notified to the sponsor within 3 days and then to the relevant parties and centers by sending the updated protocol to the investigators. Any changes must be cleared in written form and signed by all persons in charge, stating the detailed reasons for changes. If necessary, changes must be approved by the IRB and/or individual participants. A copy of the revised protocol will be added to the Investigator Site File. The protocol will be updated on the ClinicalTrials.gov registry website.

#### Dissemination plans {31a}

After the completion of the study, the results will be written as a final trial report through publication in a scientific journal along with international meetings related to neurosurgery, and the clinicaltrials.gov website.

## Discussion

The recurrence of CSDH is a major challenge, and the optimal treatment strategy to reduce the incidence of recurrence is limited although many associated risk factors have been reported [13–18]. There is solid evidence that postoperative drainage is effective in reducing symptomatic recurrence of CSDH, which is generally performed in CSDH burr-hole surgery. However, standard drainage strategy remains a matter of debate, and fixed-time drainage strategy is widely accepted, which is defined as 24 or 48 h of drainage period [8, 19–21]. Even so, according to literature, the recurrence rate is still relatively high (5–33%) with this strategy. Our observational evidence suggests that a postoperative exhaustive drainage strategy may reduce the incidence of recurrence without major complications. Thus, a randomized controlled trial is needed to evaluate the effectiveness of exhaustive drainage compared to fixed-time drainage.

In this study, we describe the rationale, design, interventions, and methodological framework of a multicenter, open-labeled, randomized controlled trial to test whether an exhaustive drainage strategy can reduce the rate of recurrence in CSDH compared with a fixed-time drainage strategy. Furthermore, we will investigate functional outcome (mRS and MGS) and quality of life (EQ-5D-5L) in the period prior to and after surgery. Moreover, the economic impact of different drainage strategies will be analyzed.

Our study has several strengths. First, our primary endpoint tests the efficacy of exhaustive drainage versus fixed-time drainage after burr-hole craniostomy in an effort to reduce the recurrence of CSDH. Second, outcome parameters that measure the functional outcome, quality of life, and healthcare-related costs are included. Third, this study will conduct in 19 neurosurgical centers in China, and all patients at 18–90 years are included. Therefore, we believe the results will be widely applicable.

In conclusion, we have developed a protocol for a multicenter, open-labeled, randomized clinical trial to evaluate the efficacy of an exhaustive drainage strategy. This study will provide an answer to whether an exhaustive drainage strategy can reduce CSDH recurrence and improve clinical outcomes, which may change clinical practice and guideline recommendations.

### Trial status

This research protocol version 1.0 (2020/07/31) is approved, and recruitment of patients for this ECHO trial has begun in December of 2020 and is expected to complete in December of 2023.

## Data Availability

After the completion and following the publication of the ECHO trial, requests for data sharing will be considered by the ECHO trial Management Group.

## Abbreviations

AE: Adverse event
CSDH: Chronic subdural hematoma
CC: Coordinating center
CONSORT: Consolidated standards of reporting trials
CT: Computerized tomography
DSMB: Data safety monitoring board
EDC: Electronic data capture
ICH-GCP: International conference on harmonization-good clinical practice
IRB: Institutional review board
MGS: Markwalder grading scale
mRS: Modified rankin scale
PI: Principal investigator
SAE: Serious adverse event
TSC: Trial steering committee
